# Effects of corruption and income inequality on the reported number of COVID-19 cases and deaths: Evidence from a time series cross-sectional data analysis

**DOI:** 10.1371/journal.pgph.0001157

**Published:** 2022-11-15

**Authors:** Atikur R. Khan, Sumaiya Abedin, Md. Mosiur Rahman, Saleheen Khan

**Affiliations:** 1 Department of Management, North South University, Dhaka, Bangladesh; 2 Department of Population Science and Human Resource Development, University of Rajshahi, Rajshahi, Bangladesh; 3 Department of Economics, Minnesota State University, MN, United States of America; Public Health Foundation of India, INDIA

## Abstract

Corruption-income inequality nexus is likely to affect the healthcare services, which in turn affect a country’s ability to suppress an epidemic. Widespread corruption in public sectors may influence the data inventory practices to control the recording and sharing of official statistics to avoid political disturbance or social problems caused by an epidemic. This empirical study examines the effects of income inequality, data inventory, and universal healthcare coverage on cross-country variation in reported numbers of COVID-19 cases and deaths in the presence of corruption in public sectors. Daily numbers of COVID-19 cases and deaths of selected 29 countries are integrated for the first 120 days of the epidemic in each country. COVID-19 dataset is then integrated with a dataset of different indices. Fixed effect panel model is applied to explore the effects of corruption perception, income inequality, open data inventory practice, and universal health coverage on the daily numbers of COVID-19 cases and deaths per million. Income inequality, corruption perception and open data inventory are found to significantly affect the number of confirmed cases and deaths. Countries with alarming income inequality are found to report 39.89 more COVID-19 cases per million, on average. Under a lower level of corruption, countries with lower level of open data inventory are expected to report 74.31 more COVID-19 cases but 1.43 less deaths per million. Given a higher level of corruption, countries with lower level of open data inventory are expected to report lower number of COVID-19 cases and deaths. Corruption demonstrates a significant influence on the size of the epidemic in terms of the number of COVID-19 cases and deaths. A country with higher level of corruption in public sector along with lower levels of open data inventory is expected to report lower number of COVID-19 cases and deaths.

## Introduction

Within the first few weeks of the reported outbreak in China, COVID-19 transmission became a global threat and the World Health Organization (WHO) officially declared a pandemic on March 13, 2020. Population level social distancing, testing and isolation strategies were the commonly adopted interventions to suppress the transmission. Both the government’s control and public compliance affect the transmission, however, the transmission continued when these intervention mechanisms are relaxed. Institutional and demographic characteristics affect public compliances and behavioural responses of citizens to any stringent policies adopted by the government [1]. This paper aims to explore the effects of institutional and demographic characteristics on cross-country variation in reported COVID-19 cases and death rates.

Though people have adopted quality steps in social distancing, early studies on spread of SARS-CoV-2 in Italy and New York provide some insight on systematic differences in regions with high median income [2, 3]. Economic position and social inequality are also associated with the level of trust in the healthcare systems [4], which in turn affects the testing rates, case counts, and data for infected individuals. Inequality fosters corruption and corruption raises inequality [5, 6]. Thus the corruption and inequality vary with each other. A few studies have shown that an increase in corruption increases the Gini coefficient of income inequality [7, 8]. A greater stringency policy is found to strongly associated with lower mortality growth during the first phase of the pandemic in countries with higher GNI per capita, higher health expenditures, and a superior level of democracy [1]. Thus, the heterogeneous effect of stringency policy is likely to vary based on cross-country differences in income inequality, good governance, and quality of universal healthcare.

It is highly likely that inequality and corruption have a bidirectional causal relationship. United Nations has also rung the alarming bell on corruption during the time of COVID-19 pandemic as the corruption affects a country’s ability to provide extended healthcare services and creating supportive environment for widespread testing and isolation [9]. Consequently, any public sector corruption in turn affects the stringency policy of a country to fight against COVID-19 epidemic. Governments with higher degrees of public sector corruption have more fear of significant political disturbance due to economic and social problems. In non-democratic countries with a lack of good governance, governments either try to limit the dissemination of negative views or manipulate the self-reported statistics to exercise damage control [10, 11]. Under-counting of number of COVID-19 deaths and cases has been discussed in some studies and the effect of such under-counting on statistical analyses has also been discussed [12]. Application of the last-digit test has demonstrated the existence of such data manipulation and malpractice in releasing official statistics both in a democratic and authoritarian regime [13]. Thus data inventory practice (complete record keeping and management of datasets for storing and sharing) may play important role in the cross-country variation in officially reported numbers of COVID-19 cases and deaths.

Quality of official statistics largely depends on the practice of good governance and the level of corruption in public sectors. For a greater transparency in administration, governments release some data as open data for public use. Open data refers to data that are made available to public free of cost without any restriction of use, re-use, and redistribution. Sharing open data as public good enhances citizen participation in innovation and policymaking during an emergency. The main source of information regarding the size, severity, and control strategies for an epidemic is the national statistics and COVID-19 related national statistics are disseminated as open data for public good. However, data inventory practice and dissemination of national statistics as open data can be affected by many factors especially by the government’s goodwill and transparency. Open data inventory is deemed to be a crucial tool to enable a culture of transparency, accountability, and access to information to effectively reduce the corruption in public sectors [14]. In the presence of corruption and an authoritarian regime, open data quality is likely to be compromised. Thus, the corruption-income inequality nexus is deemed to affect the universal health coverage and data quality. The present study therefore hypothesises that: corruption level deteriorates the universal health coverage and increases both the number of COVID-19 infections and deaths; lower degree of income inequality enhances the universal health coverage and reduces the COVID-19 infection and deaths; and higher level of corruption worsens the quality of disseminated data which in turn affects the reported COVID-19 infection and death rates.

## Materials and methods

### Data processing and integration

For a data-driven exploration of the hypotheses, we integrate net income Gini index (NIGI), corruption perception index (CPI), universal health coverage index (UHCI), and open data inventory index (ODINI) data with COVID-19 data [15–18]. We have obtained COVID-19 data of 210 countries from https://ourworldindata.org. Since the time to report the first COVID-19 confirmed case is not unique for all countries, we consider the first 120 days data from the date of the first confirmed case in each country. As of 31 July 2020, average numbers of confirmed cases across all countries in the first 120 days are computed and countries exceeding this average number of COVID-19 cases are included in this study. This filtering approach for country selection excludes countries with too low number of reported cases. Too low numbers of reported cases are due to some systematic differences of stringency policy, testing and reporting strategies. Accordingly, the adopted filtering procedure enables us to avoid too many zero counts ensuring sufficient counts for confirmed number of infections (reported confirmed cases) and deaths in daily time series. Daily time series of COVID-19 data of selected 34 countries are then integrated with UHCI, CPI, NIGI, and ODINI data sets.

From the list of selected 34 countries, Bangladesh has been excluded due to systematic difference in stringency policy with limited number of selective tests and upfront payment requirement for COVID-19 tests [19]. Because of missing information on some indices, Kuwait has also been excluded from this cross-country study. Three other countries (Ecuador, Portugal and Spain) have been excluded from the study because of negative counts of cases and deaths due to the revision of COVID-19 official statistics by these countries. Thus we form a panel data frame with COVID-19 cases and deaths per million of population for the first 120 days from the first confirmed case in each of these 29 countries.

**Fig 1:**
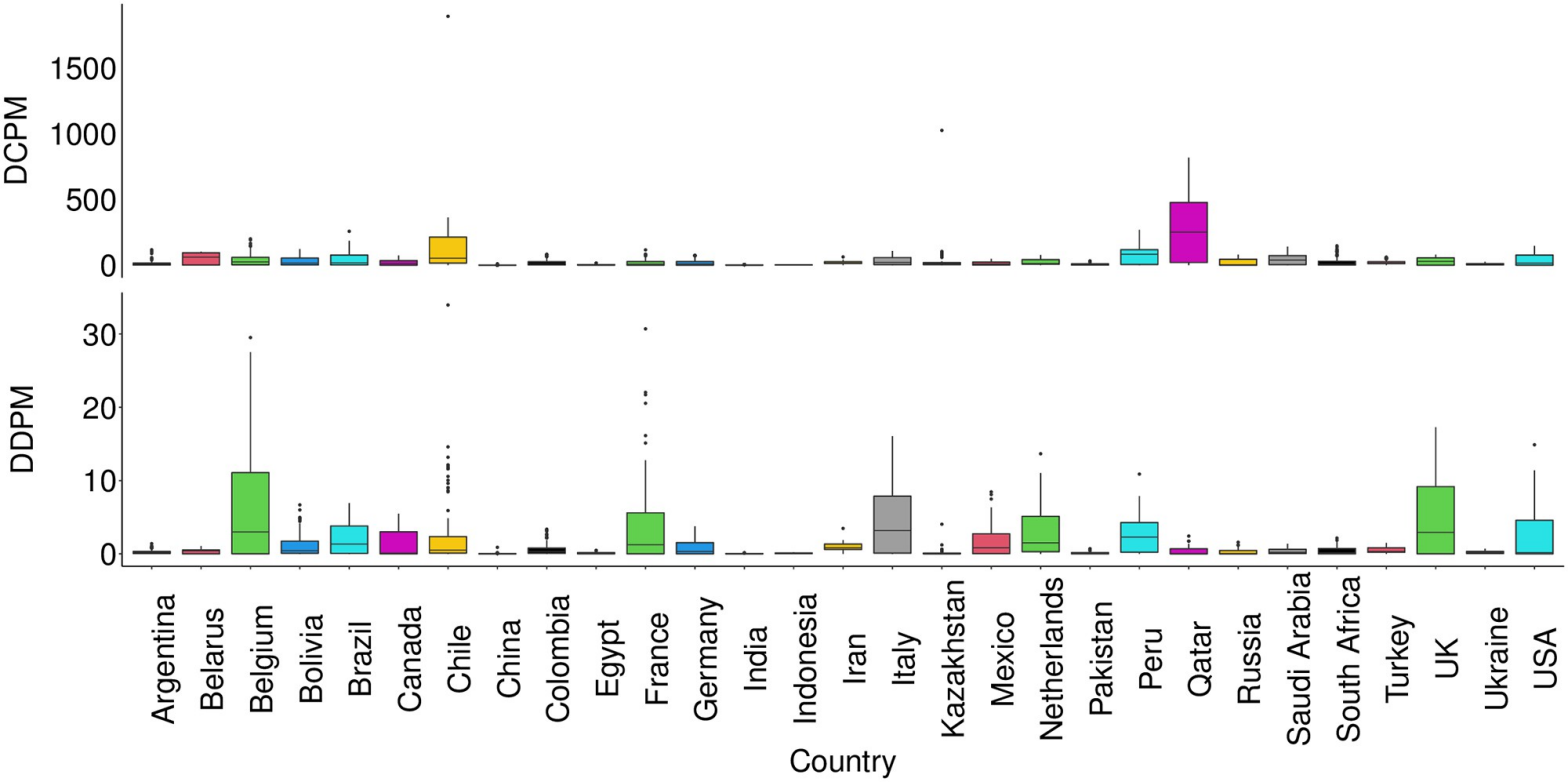
Distribution of number of cases (DCPM) and deaths (DDPM) per million of population.

COVID-19 epidemic started in China and turned into a pandemic within months. Most of the countries had to start containing this epidemic with the existing healthcare systems and many countries had to wait for international aids to improve the healthcare services to fight against the epidemic [20]. So, the very first phase of the epidemic would better reflect the governance and management of healthcare services of a country. As the time passes, variation of a time series is characterized mainly by the autocorrelation and time dependent factors (temperature, humidity, etc.) by making time invariant factors (corruption, population density, etc.) insignificant [21, 22]. Consequently, sprouting different variants of SARS-CoV-2 (Delta, Alpha, and Omicron) over time is infusing more heterogeneity in data. Thus a relatively shorter time series around the first phase of the epidemic is deemed to better infer the effects of corruption perception in public sector, income inequality, healthcare coverage, and so on. So, we have considered the first 120 days of the epidemic in each of these selected countries to construct a panel data frame. Distribution of daily confirmed cases and deaths per million of population of these selected countries are shown in Fig 1.

### Statistical analysis

We would like to explore the effects of some key indicators on daily number of cases per million of population. Thus we apply fixed effect panel regression model for both daily number of cases per million (DCPM) and for daily number of deaths per million (DDPM) in response to the exogenous variables CPI, NIGI, UHCI, and ODINI. To avoid the multicollinearity problem in panel data regression with these exogenous variables, we have examined the variance inflation factor (VIF). Since the VIF for CPI, NIGI, ODINI and UHCI are 1.7514, 1.3126, 1.6096, and 1.7073 respectively, therefore, none of the exogenous variables are excluded from the analyses [23].

The NIGI is a measure for net income inequality and the index ranges between 0 and 1 where a value 0 stands for perfect equality and a value of 1 refers to perfect inequality. A value of NIGI above 0.40 is considered alarming level of inequality [24]. Thus a panel regression model can be employed to explore the effect of alarming level of inequality on COVID-19 cases and death rates compared to a non-alarming level of income inequality. This can be done effectively by constructing a dichotomous variable of the form: alarming level of inequality (*NIGI* ≥ 0.40) and non-alarming level of inequality (*NIGI* < 0.40). Similarly, we can construct dichotomous variables for UHCI, ODINI and CPI.

The CPI reflects the perceived level of public sector corruption and it ranges between 0 and 100 with 0 meaning highly corrupt and 100 meaning very clean [16]. Corruption involves illegal and hidden activities which only come to light through scandals or prosecutions. Thus it is not possible to quantify the level of corruption in public sectors by collecting data based only on scandals or prosecutions. The Transparency International (TI) has computed CPI based on carefully designed and calibrated questionnaires on public sector corruptions (bribery, diversion of public funds, officials using their public office for private gain, ability of government to contain corruption in public sector, and so on) answered by experts and businesspeople [16]. Thus it measures how corrupt each country’s public sector is perceived to be, according to experts and businesspeople. However, the composite score CPI portrays the presence of public sector corruption in a country and serves as an indirect measure of public sector corruption to perform a cross-country comparison on a 0 − 100 scale (0 means highly corrupt and 100 means very clean).

The UHCI is the UHC Service Coverage index that is also the official measure of sustainable development goal (SDG) indicator. This index ranges between 0 and 100 with the value 100 meaning the best level of UHC [17]. An ODIN index is a global measure that reflects the openness and coverage of official statistics [18]. This score ranges between 0 and 100 with higher scores indicating the higher level of coverage and openness in official statistics. The global average of CPI is 43 and that for UHCI is 66 [16, 17]. On the other hand, the global median is 41.1 for ODIN scores [18]. Akin to the process of categorizing variables for inclusion in a statistical model, we also categorize CPI, UHCI, and ODINI as low for any score less than the global average (or median) and high for any score more than or equal to the global average (or median). Thus the exogenous variables considered in our panel regression model are: CPI (Low: Score < 43, High: Score ≥ 43), UHCI (Low: Score < 66, High: Score ≥ 66), ODINI (Low: Score < 41.1, High: Score ≥ 41.1), and NIGI (Non-alarming: Score < 0.40, Alarming: Score ≥ 0.40).

Higher population density is expected to increase the likelihood of spreading the virus which in turn may result in higher number of COVID-19 cases per million [25–27]. Thus this variable is also included in the model to adjust the effect of other exogenous variables.

## Results

**Fig 2:**
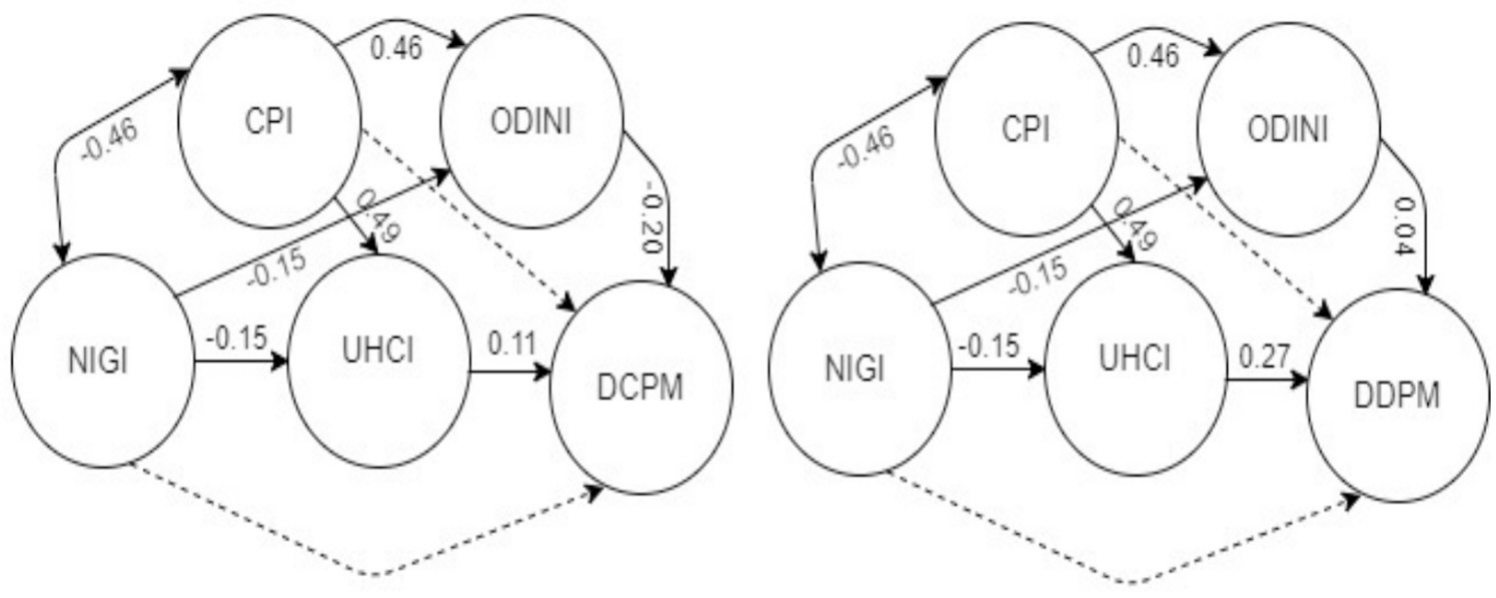
Empirical relationships between indices and COVID-19 cases (left panel) and deaths (right panel) with different pathways.

As has been explored in the previous sections, the income inequality and corruption perception likely to have a bidirectional causal relationship. We also have hypothesized that the corruption-income inequality nexus affects the universal healthcare and official data dissemination which in turn affects the reported number of COVID-19 cases and deaths. Since the statistical analysis does not support the existence of multicollinearity in the data, we can utilize the panel model to examine the relationships among the variables by constructing a path diagram shown in Fig 2.

Results from the path model demonstrate that the effects of NIGI and CPI are mediated through the variables UHCI and ODINI. We also have explored earlier that there is no multicollinearity in the data. Thus we apply panel models to examine the effects of these variables. Coefficients of fixed effect models for *DCPM*_*i*,*t*_ and *DDPM*_*i*,*t*_ are provided in Table 1 where the population density is found to significantly affect the number of daily reported cases.

**Table 1:** Fixed effect estimate of parameters for panel models of daily number of cases (DCPM) and death (DDPM) per million of population.

	DCPM		DDPM	
Variables	Estimate	p-value	Estimate	p-value
**Population density**	0.0599	0.0001	0.0049	0.0001
**CPI Levels:**				
High (CPI ≥43)	34.7358	0.0001	0.9106	0.0001
Low (CPI <43)	—		—	
**UHCI Levels:**				
High (UHCI ≥66)	40.3124	0.0001	1.742	0.0001
Low (UHCI <66)	—		—	
**NIGI Levels:**				
Alarming (NIGI ≥40)	39.8928	0.0001	-0.2735	0.0099
Not Alarming (NIGI <40)	—		—	
**ODINI Levels:**				
High (ODINI ≥41.1)	-38.536	0.0001	0.8418	0.0001
Low (ODINI <41.1)	—		—	

Any p-value less than 0.0001 is presented as 0.0001. Higher CPI refers to lower level of corruption, and an increase in CPI refers to a decrease in public sector corruption.

It seems that for one more person per square kilometre increases the number of COVID-19 cases by 0.0599 per million. This finding aligns with the exposition that the higher population density is likely to increase the likelihood of spreading the virus that in turn may result in higher number of COVID-19 cases [25–27]. The inclusion of this variable has adjusted the effect of other variables. On average, countries with the higher level of CPI (lower degree of corruption) report 34.72 more COVID-19 cases and 0.91 more deaths per million of population compared to countries with higher level of corruption (low CPI). Similarly, countries with higher level of universal health coverage (higher UHCI) are expected to have 40.31 more reported cases and 1.74 more deaths per million when compared to the countries with lower level of universal health coverage. It seems that both the number of reported COVID-19 cases and deaths are lower for countries having higher level of corruption and lower level of universal health coverage.

Both income inequality and open data inventory are found to affect the number of confirmed cases and deaths. Countries with alarming level of income inequality are not only expected to report 39.89 more COVID-19 cases per million but also expected to report less number of confirmed COVID-19 deaths per million. Countries with higher level of openness and coverage of official statistics have 38.57 less COVID-19 cases compared to the countries with lower level of ODIN scores, on average. However, countries with higher level of ODIN scores are found to have higher number of COVID-19 deaths. This is an indication that a country with rich data inventory is likely to report more cases and deaths with higher degrees of openness.

We have explored in Fig 2 that the effect of CPI on COVID-19 cases is mediated through the UHC. Thus we explore the joint effect of CPI and UHCI in a fixed effect model in Table 2.

**Table 2:** Fixed effect estimate of interactions between CPI and UHCI levels, and ODINI and NIGI levels.

	DCPM		DDPM	
Variables	Estimate	p-value	Estimate	p-value
**Population Density**	0.0416	0.0002	0.0050	0.0001
**(CPI, UHCI) Levels:**				
(High, High)	—		—	
(Low, High)	-24.8604	0.0001	-0.9811	0.0001
(Low, Low)	-50.7680	0.0001	-2.8260	0.0001
**(ODINI, NIGI) Levels:**				
(High, Not Alarming)	—		—	
(High, Alarming)	19.7442	0.0001	-0.1296	0.3583
(Low, Not Alarming)	6.7619	0.1590	-0.6149	0.0010
(Low, Alarming)	85.7619	0.0001	-1.1677	0.

Any p-value less than 0.0001 is presented as 0.0001. High and Low categories are as in Table 1. A higher CPI refers to a lower corruption and vice versa.

Results in Table 2 demonstrate that a country with higher level of corruption (lower CPI score) and lower level of universal health coverage (lower UHCI score) is expected to report 50.77 less cases and 2.83 less deaths per million of population compared to a country with lower level of corruption and higher level of universal health coverage. When compared to a country with lower level of corruption and higher level of UHC, a country with higher level of corruption even with higher level of UHC is expected to have 24.86 less confirmed COVID-19 cases and 0.98 less COVID-19 deaths per million.

The path diagram in Fig 2 depicts the effect of CPI on COVID-19 cases and deaths via the ODIN. Thus we explore the interaction effects of CPI and ODINI (interaction of corruption level with openness and coverage of official statistics) and UHCI and NIGI (interaction of universal health coverage and income inequality) on the daily numbers of COVID-19 cases and deaths. Results of fixed effect model for these interaction terms are provided in Table 3.

**Table 3:** Fixed effect estimate of interactions between NIGI and UHCI, and CPI and ODINI levels.

	DCPM		DDPM	
Variables	Estimate	p-value	Estimate	p-value
**Population Density**	0.0856	0.0001	0.0047	0.0001
**(CPI, ODINI) Levels:**				
(High, High)	—		—	
(High, Low)	74.3159	0.0001	-1.4296	0.0001
(Low, High)	-9.0508	0.0150	-1.2742	0.0001
(Low, Low)	-20.0205	0.0002	-1.7768	0.0001
**(UHCI, NIGI) Levels:**				
(High, Not Alarming)	—		—	
(High, Alarming)	27.5322	0.0001	0.0371	0.7663
(Low, Not Alarming)	-5.8543	0.4829	-1.1963	0.0003
(Low, Alarming)	-26.4394	0.0001	-1.9892	0.0001

Any p-value less than 0.0001 is presented as 0.0001. High and Low categories are as in Table 1. A higher CPI refers to a lower corruption and vice versa.

Among the countries with non-alarming level of income inequality (lower level of inequality), a country with lower level of UHC is expected to have lower number of confirmed cases and deaths compared to a country with higher level of UHC. Compared to countries with higher level of UHC and non-alarming level of inequality, a country with lower level of UHC and higher level of inequality is likely to expect 26.44 less COVID-19 cases and 1.99 less deaths per million. This contradicts with the usual assumption that a country with higher level of corruption and income inequality will experience more COVID-19 cases and deaths per million. Data inventory practice may cause this differential effect and such effect can be explored through the interaction effects of ODINI and CPI shown in Table 3.

Among the low corruption countries (countries with higher values for CPI), a country with lower level of openness and coverage of official statistics (lower ODIN index) is expected to obtain higher number of reported cases (74.31 more cases per million) for COVID-19 infections but lower number of COVID-19 related deaths (1.43 less death per million). Among the countries with higher level of data openness and coverage, high corruption countries are expected to have lower number of cases and deaths. Among the high corruption countries (countries with lower level of CPI), a country with lower level of data openness and coverage is expected to report lower number of COVID-19 cases and deaths per million. Moreover, corruption and open data inventory nexus strongly affect the cross-country variation in daily number of COVID-19 cases and death rates.

## Discussion

Corruption, income inequality, universal health coverage, and openness and coverage of official statistics are assumed to affect the number of cases and deaths per million of a population. Higher level of income inequality is expected to negatively affect the implementation of any suppression intervention via social distancing, working from home, masking, testing, and isolation. Lower level of income inequality with higher level of universal healthcare service coverage is expected to provide better support to citizens for life saving medical treatment along with the testing, isolation and social distancing to reduce the transmission. Surprisingly, our empirical study reveals that higher number of COVID-19 cases and deaths per million of population are reported in countries with higher level of universal health coverage and lower level of income inequality.

Corruption may directly affect and deteriorate healthcare services, which in turn may affect the services to reduce transmission and provide services to COVID-19 patients. Thus high corruption in public sector along with a low level of universal health coverage is expected to surge the number of cases and deaths. However, this is not supported by our empirical research, rather a highly corrupted country with low level of universal health coverage is found to have lower numbers of reported cases and deaths compared to a low corruption country with high level of universal health coverage. In the presence of higher level of corruption in public sectors, a country with low level of openness and coverage of official statistics is expected to report a smaller number of cases and deaths compared to a country with higher level of openness and coverage for official statistics. Moreover, high levels of corruption and low levels of openness and coverage of official statistics reduce the number of reported cases and deaths per millions of people. Hence, the corruption and open data inventory nexus seems to have played a role in the cross-country variation of reported number of COVID-19 cases and deaths per million.

### Limitation of the study

Our empirical study has found significant effects of CPI and ODINI on the size and severity of COVID-19. Though CPI is a measure for corruption perception, it reflects the existence of corruption in public sectors and does not necessarily manifest the occurrence of corruption in containing the COVID-19 epidemic. Similarly, ODINI is a measure for openness and coverage of official statistics that does not reflect the data quality compromised by the human factors or data processing systems. The ODINI score does not manifest any malpractice in manipulating COVID-19 data and should be carefully interpreted. Since corruption involves illegal and hidden activities, there is no data to demonstrate that inventory practices have been compromised because of corruptions happened during the management of COVID-19 epidemic. Nevertheless, both CPI and ODINI jointly provide an insight regarding the existence of corruption in public sectors and openness and coverage of COVID-19 statistics to explore how these measures affect the variation of the size and severity of the epidemic.

## Conclusion

Corruption in public sector, income inequality and open data inventory significantly influence the reported size and severity of an epidemic. The less the public sector corruption, the more the size and severity of a reported epidemic in the presence of higher degrees of open data inventory. Alarming level of income inequality and lower level of open data inventory increases the reported size but reduces the reported severity of an epidemic.
